# Lipidomic analysis coupled with machine learning identifies unique urinary lipid signatures in patients with interstitial cystitis/bladder pain syndrome

**DOI:** 10.1007/s00345-025-05628-y

**Published:** 2025-04-18

**Authors:** Takuya Iwaki, Makoto Kurano, Masahiko Sumitani, Aya Niimi, Akira Nomiya, Jun Kamei, Satoru Taguchi, Yuta Yamada, Yusuke Sato, Masaki Nakamura, Daisuke Yamada, Tomonori Minagawa, Hiroshi Fukuhara, Haruki Kume, Yukio Homma, Yoshiyuki Akiyama

**Affiliations:** 1 Department of Urology, Graduate School of Medicine, The University of Tokyo, Tokyo, Japan; 2 Department of Urology, Chiba Tokushukai Hospital, Chiba, Japan; 3 Department of Clinical Laboratory Medicine, The University of Tokyo, Tokyo, Japan; 4 Department of Pain and Palliative Medicine, The University of Tokyo Hospital, Tokyo, Japan; 5 Department of Urology, Japan Organization of Occupational Health and Safety, Kanto Rosai Hospital, Kanagawa, Japan; 6 Department of Urology, Tokyo Metropolitan Tama Medical Center, Tokyo, Japan; 7 Department of Urology, NTT Medical Center Tokyo, Tokyo, Japan; 8 Department of Urology, Shinshu University School of Medicine, Nagano, Japan; 9 Department of Urology, Kyorin University School of Medicine, Tokyo, Japan; 10 Department of Interstitial Cystitis Medicine, Kyorin University School of Medicine, Tokyo, Japan

**Keywords:** Interstitial cystitis, Bladder pain syndrome, Urinary, Biomarker, IC/BPS, IC, Hunner, Lipidomics, Machine, Learning

## Abstract

**Purpose:**

To identify biomarkers for diagnosis and classification of interstitial cystitis/bladder pain syndrome (IC/BPS) by urinary lipidomics coupled with machine learning.

**Methods:**

Urine samples from 138 patients with IC/BPS, including 116 with Hunner lesion (HL) and 22 with no HL, and 71 controls were assessed by lipid chromatography-tandem mass spectrometry. Single and paired lipid analyses of differentially expressed lipids in each group were conducted to assess their diagnostic ability. Machine learning models were constructed based on the identified urinary lipids and patient demographic data, and a five-fold cross-validation method was applied for internal validation. Levels of urinary lipids were adjusted to account for urinary creatinine levels.

**Results:**

A total of 218 urinary lipids were identified. Single lipid analysis revealed that urinary levels of C24 ceramide and LPC (14:0) distinguished HL and no HL, with an area under the receiver operating characteristics curve of 0.792 and 0.656, respectively. Paired lipid analysis revealed that summed urinary levels of C24 ceramide and LPI (18:3), and subtraction of PG (36:5) from PC (38:2) distinguished HL and no HL even more accurately, with an area under the curve of 0.805 and 0.752, respectively. A machine learning model distinguished HL and no HL, with the highest area under the curve being 0.873 and 0.750, respectively. Limitations include the opaque black box nature of machine learning techniques.

**Conclusions:**

Urinary levels of C24 ceramide, along with those of C24 ceramide plus LPI (18:3), could be potential biomarkers for HL. Machine learning-coupled urinary lipidomics may play an important role in the next-generation AI- driven diagnostic systems for IC/BPS.

**Supplementary Information:**

The online version contains supplementary material available at 10.1007/s00345-025-05628-y.

## Introduction

Interstitial cystitis/bladder pain syndrome (IC/BPS) is an intractable, enigmatic disorder characterized by persistent bladder pain and urinary frequency and urgency, which have a severe impact on patients’ quality of life (QOL) [1]. The detailed pathophysiologies of IC/BPS are still unknown, and so standardized diagnostic criteria and curable treatments have yet to be developed. Growing evidence, however, has helped us to understand a clearer picture of IC/BPS, which comprises at least two distinct disorders: Hunner-type IC (HIC) that is characterized by the cystoscopic presence of a Hunner lesion in the bladder and BPS that lacks a Hunner lesion [2]. Recent studies reveal that HIC and BPS are distinct entities with different pathophysiologies: the former is an immune-mediated inflammatory disease with a possible autoimmune component, and the latter is a non-inflammatory symptom syndrome with little obvious bladder pathology and comprised of multiple clinical phenotypes [3–8]. Thus, treatment strategies are different between patients with HIC and those with BPS. For patients with HIC, the Hunner lesion-targeted treatments such as fulguration of the lesions and intravesical injection of triamcinolone into the lesions, in conjunction with systemic immunomodulatory therapies using cyclosporine A and prednisolone for intractable cases, are recommended [9]. Meanwhile, multidisciplinary management including pain control, physiotherapy, systemic neuromodulation, and coping with psychosocial problems are recommended for patients with BPS [1]. Therefore, the precise diagnosis of each subtype is indispensable for optimal clinical management of IC/BPS. Based on this, cystoscopy at initial evaluation of patients suspected of having IC/BPS is crucial since the cystoscopic presence of Hunner lesions is the only diagnostic criterion for HIC; however, the cystoscopic appearance of Hunner lesions varies between cases, and no objective diagnostic markers have been established. This renders a precise diagnosis of Hunner lesions a highly complicated process for general urologists. Thus, reliable, objective, and less invasive disease markers of IC/BPS are an urgent unmet clinical need.

Different molecular species of lipids are distributed throughout all body tissues, playing a pivotal role in cellular functions such as signal transduction, transformation of cell membranes, formation of molecular scaffolds, and supply of mitochondrial cellular bioenergetics [10]. Bioactive lipid molecules such as lysophospholipids (LPLs), ceramides, and eicosanoids are involved in various diseases such as diabetes mellitus, cardiovascular disease, and some malignancies [11, 12]. Specifically, recent studies show that these bioactive lipids play a crucial role in inflammatory processes by inhibiting inflammatory cell infiltration, increasing clearance of apoptotic cells, and stimulating mucosal antimicrobial defenses [13]. Previously, we measured the urinary levels of selected bioactive lipids in patients with acute bacterial cystitis, including six ceramides, sphingosine (Sph), dihydrosphingosine (dhSph), sphingosine 1-hosphate (S1P), and dihydrosphingosine 1-phosphate (dhS1P). We found that urinary levels of LPL mediators such as lysophosphatidylcholine (LPC), lysophosphatidylethanolamine, and sphingolysophospholipids were elevated in patients with acute bacterial cystitis [14]. These results indicate a possible association between these urinary lipid molecules and inflammatory processes in the bladder; however, the full picture regarding the relationship between urinary lipid signatures and bladder inflammation remains enigmatic since large-scale, comprehensive measurement of the immense number of the whole lipid species is technically difficult. Recent technological advances, namely lipidomic analysis or lipidomics, have enabled simultaneous quantification and mapping of entire cellular lipid molecular species, based on mass spectrometry, an analytical technique used to measure the mass-to-charge ratio of ions [10, 15].

The aim of the present study was to use lipidomic analysis to obtain the whole urinary lipid signatures in patients with IC/BPS, and to identify urinary lipid molecules involved specifically in these conditions. In addition, we used machine learning algorithms to analyze the immense lipidomic data, and developed an artificial intelligence (AI)-driven urinary diagnostic models for IC/BPS.

## Materials and methods

### Ethics statement

The study was approved by the Institutional Review Board of the University of Tokyo, including the use of an opt-out method to obtain informed consent (approval no. 2602 and 3124). Patients were informed about the study protocol using generally accessible contact information, and those who chose to take part provided written informed consent. All procedures followed appropriate ethical guidelines for clinical studies.

### Sample collection and patient demographics

Spot midstream urine samples were collected from 116 patients with HIC, 22 patients with BPS, and 71 control patients; samples were obtained during outpatient visits to the urology clinic at the University of Tokyo Hospital from September 2020 to March 2021. Diagnosis and classification of IC/BPS was based on the East Asian clinical guidelines [2]. Patient symptoms (at the time that the samples were collected) were assessed using the IC/BPS symptom scores measured by the O’Leary and Sant symptom index (OSSI) and problem index (OSPI) [16]; an 11-point numerical rating scale of pain intensity, with 0 indicating no pain and 10 indicating the worst pain ever; a frequency volume chart including the daytime and nighttime urinary frequency; and the average and maximum voided volume. All patients with HIC had been treated with transurethral resection of Hunner lesions with concomitant bladder hydrodistension, and were regularly followed up during outpatient visits. These patients managed symptom flairs using only analgesia and antibiotics. Patients with BPS were treated with oral medications on an outpatient basis. None of the patients with IC/BPS had received intravesical therapies before enrollment. Control urine samples were obtained from 71 patients with benign urological diseases (*n* = 28) or a previous history of urological malignancy (*n* = 43); samples were obtained at outpatient visits (as for patients with IC/BPS). All control patients were free from bladder pain and discomfort. Collected urine samples were stored at -80 °C until measurement were conducted, and all samples were handled on ice during the procedures (except during necessary incubation processes). Further details are given in the Supplement.

### Measurement of urinary lipids

Urinary levels of 413 lipid species, including lysophosphatidic acid, lysophosphatidylcholine, lysophosphatidylethanolamine, lysophosphatidylglycerol, lysophosphatidylinositol (LPI), lysophosphatidylserine (LPS), dhSph, Sph, phosphatidylcholine (PC), phosphatidylethanolamine (PE), phosphatidylglycerol (PG), phosphatidylinositol (PI), phosphatidylserine (PS), sphingomyelin (SM), and ceramide were quantified simultaneously by liquid chromatography-mass spectrometry (LC-MS) (Shimazu, Japan). Urinary levels of the measured lipids were adjusted to account for urinary creatinine levels.

### Differentially expressed lipid (DEL) analysis and the disease classification test

DELs between the three groups were searched based on the log_2_-normalized urinary lipid levels by the Kruskal–Wallis test followed by the Benjamini–Hochberg correction. The statistical significance of differences in urinary lipid levels were evaluated at a false discovery rate (FDR) < 0.05. Then, group-specific DEGs were identified by the Steel–Dwass post hoc analysis with the statistical significance set at *p*-value < 0.05 and *log*_*2*_*[Ratio]* ≥ 1 in pairwise comparisons between groups. The disease classifier ability of each identified group-specific DEL was tested using multiple linear and logistic regression analyses (single lipid analysis). Nonparametric receiver operating characteristic (ROC) curves for the urinary level of each group-specific DEL were then created. The area under the curve (AUC) was calculated using a bootstrap method, with 1,000 iterations for the 95% confidence intervals. The cut-off value for the urinary lipid levels with the highest AUC was determined by the maximum Youden index [17]. Next, the disease classifier ability was tested further by performing arithmetic operations (i.e., addition, subtraction, multiplication, and division) on every pair of DELs in each group (paired lipid analysis). Additional variables were created by performing arithmetic operations on each pair of group-specific DELs. Disease classification tests for these explanatory variables were conducted using ROC analysis, as described above.

### Application of machine learning to disease classifier models based on multiple DELs

PyCaret (https://www.pycaret.org/tutorials), a low-code automated machine learning library for Python that enables simultaneous application of multiple different algorithms was used to develop disease classifier machine learning models based on multiple urinary lipids in conjunction with patient demographic data (age and sex). The Extreme Gradient Boosting (XGBoost) classifier model was applied to the machine learning framework [18]. Patient data were divided randomly into training and test datasets at a 7:3 ratio, with an equivalent proportion of each of the three groups in both datasets. Five-fold cross-validation was conducted to optimize the hyperparameters of a model in the training dataset, and classification performance was evaluated for the test dataset. In the present study, eight models were developed to account for variations in the input lipid data: all 218 lipids; DELs between the all groups identified in the FDR-corrected Kruskal–Wallis test (threshold, FDR < 0.05); DELs identified at a threshold of FDR < 0.01; and DELs identified at a threshold of FDR < 0.001. These four-group parameters were subjected to machine learning analyses with or without patient demographic data (i.e., age and sex), comprising a total of eight models. The AUC was calculated for each model, along with micro-averaged and macro-averaged AUCs to summarize overall performance across all models [19].

### Statistical analysis

Statistical analyses of urinary lipid data processing and machine learning analyses were conducted using the Scikit-learn library in Python [20]. Other statistical analyses were carried out using JMP^®^ Pro software, ver. 11 (SAS Institute, Cary, NC). Further details are given in the Supplement.

## Results

### Characteristics of the study subjects

Patient demographics are shown in Table 1. Patients with HIC had a significantly smaller maximum voided volume and greater nighttime urinary frequency than those with BPS, while pain intensity was significantly higher in patients with BPS than in those with HIC. There were no significant differences in the OSSI/OSPI scores, daytime urinary frequency, and average voided volume between patients with HIC and BPS. Medications and comorbidities at the time of sample collection are listed in Tables 2 and 3, respectively.

**Table 1:** Demographics of the study subjects

					*P*-value​^†^	
	HIC	BPS	control	HIC vs. BPS​	HIC vs. control​	BPS vs. control​
No. of patients	116	22	71			
Age​ (years)	69.9 ± 10.9 [37–86]. ​^‡^	60.1 ± 13.5​ [29–80]	71.5 ± 12.1 [21–94]	< 0.01*	0.14	< 0.01*​
Sex (male/female)​	19/97	2/20​	48/23​	1.0	< 0.01​*	< 0.01​*
OSSI	10.8 ± 4.7 [1–20]	8.6 ± 4.1 [1–17]	NA	0.38	NA	NA
OSPI	8.0 ± 4.4 [0–16]	8.6 ± 4.1 [1–12]	NA	0.62	NA	NA
Day time frequency	11.5 ± 5.7 [5–43]	11.0 ± 4.1 [6–19]	NA	1.0	NA	NA
Night time frequency	3.6 ± 2.1 [0–9]	1.3 ± 1.3 [0–4]	NA	< 0.01*	NA	NA
Average voided volume (mL)	126.8 ± 68.2 [30–400]	147.3 ± 66.1 [50–255]	NA	0.22	NA	NA
Maximum voided volume (mL)	209.1 ± 146.5 [45–800]	268.2 ± 106.3 [110–400]	NA	0.03*	NA	NA
Pain intensity	3.2 ± 2.8 [0–10]	5.2 ± 2.9 [0–8]	NA	0.03*	NA	NA

NA: not applicable

^†^Wilcoxon rank sum test for two-group comparisons and Steel–Dwass test for three group comparisons for continuous variables, or Fisher’s exact test for categorical variables

*Statistical significance: *p* < 0.05 in the Steel–Dwass test or Fisher’s exact test for pairwise comparison between groups

^‡^Mean ± SD [range]

BPS: Bladder pain syndrome; HIC: Hunner-type interstitial cystitis; OSSI/OSPI: O’Leary and Sant symptom index/O’Leary and Sant problem index

**Table 2:** Medications

	HIC (*n* = 116)	BPS (*n* = 22)	control (*n* = 71)	*P*-values
Antihypertensive drugs	26 (22.4)	1 (4.5)	22 (31.0)	0.04*
Diabetes medication	6 (5.2)	1 (4.5)	9 (12.7)	0.15
Lipid-lowering drugs	20 (17.2)	3 (13.6)	15 (21.1)	0.67
Urate-lowering drugs	1 (0.9)	1 (4.5)	4 (5.6)	0.15
Anticholinergics	6 (5.2)	1 (4.5)	7 (9.9)	0.42
Beta3 agonist	13 (11.2)	2 (9.1)	11 (15.5)	0.61
Tricyclic anti-depressant	5 (4.3)	12 (54.5)	0 (0)	< 0.01*
Suplatast tosilate	2 (1.7)	5 (22.7)	0 (0)	< 0.01*
NSAIDs	18 (15.5)	0 (0)	2 (2.8)	< 0.01*
Analgesia	21 (18.1)	4 (18.2)	2 (2.8)	< 0.01*
Immunosuppressants	12 (10.3)	0 (0)	4 (4.2)	0.11
Hypnotic	25 (21.6)	12 (54.5)	7 (9.9)	< 0.01*
Other drugs	43 (37.1)	11 (50.0)	28 (39.4)	0.52
Diuretic	2 (1.7)	0 (0)	2 (2.8)	0.68
Gastrointestinal drugs	38 (32.8)	9 (40.9)	20 (28.2)	0.68
Antithrombotic drugs	10 (8.6)	4 (18.2)	11 (15.5)	0.24
Thyroid disorder medications	3 (2.6)	1 (4.5)	4 (5.6)	0.56

*Statistical significance: *p < 0.05; Fisher’s exact test

BPS: bladder pain syndrome; HIC: Hunner-type interstitial cystitis; IPD: suplatast tosilate; NSAIDs: Nonsteroidal anti-inflammatory drugs

**Table 3:** Comorbidities

	HIC	BPS	Control	*P*-value
Hypertension	38 (32.8)	3 (13.6)	29 (40.8)	0.06
Diabetes mellitus	12 (10.3)	1 (4.5)	13(18.3)	0.14
Dyslipidemia	22 (19.0)	3 (13.6)	16 (22.5)	0.63
Hyperuricemia	2 (1.7)	1 (4.5)	4 (5.6)	0.33
Carcinoma	21 (18.1)	3 (13.6)	49 (69.0)	< 0.01*
Neurological diseases	30 (25.6)	0 (0)	2 (2.8)	0.74
Autoimmune diseases	13 (11.2)	1 (4.5)	2 (2.8)	0.09
Psychiatric disorders	5 (4.3)	4 (18.2)	2 (2.8)	0.02*
Other diseases	38 (32.7)	6 (27.3)	26 (36.6)	0.70
Cardiovascular diseases	8 (6.9)	0 (0)	7 (9.8)	0.29
Cerebrovascular diseases	6 (5.2)	2 (9.1)	3 (4.2)	0.67
Respiratory diseases	5 (4.3)	1 (4.5)	6 (8.5)	0.48
Endocrine disorders	8 (6.9)	1 (4.5)	8 (11.3)	0.46
Gastrointestinal diseases	25 (21.5)	4 (18.2)	10 (14.1)	0.44

*Statistical significance: *p* < 0.05; Fisher’s exact test

BPS: bladder pain syndrome; HIC: Hunner-type interstitial cystitis

### LC-MS and identification of DELs

**Fig. 1:**
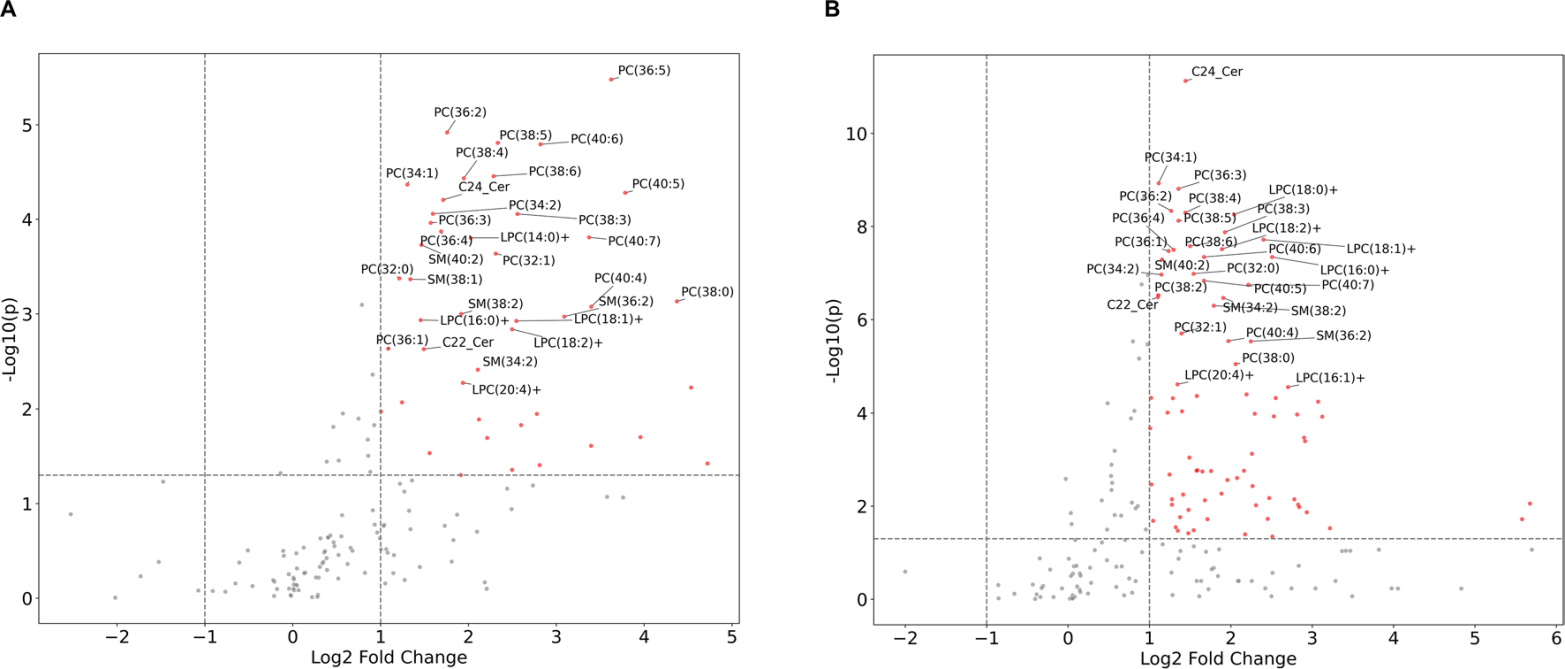
Volcano plots showing the urinary levels of different lipid species in the single lipid analysis. **A**: DELs between HIC and BPS. **B**: DELs between HIC and controls. The log2-fold change (FC) was plotted against the -log_10_*p*-value (P). Red dots indicate a *log*_*2*_*Ratio* ≥ 1

Of the 413 urinary lipid species detected in all samples, 195 were not expressed across all subjects and were therefore excluded from the study (Supplementary Table S1). The remaining 218 lipid species were subjected to further analyses. Of these, an FDR-corrected Kruskal-Wallis test identified 102 lipid metabolites differentially expressed across all groups (Supplementary Table S2). A post hoc Steel–Dwass test identified 66 DELs between the HIC and BPS groups, 100 DELs between the HIC and control groups, and three DELs between the BPS and control groups (Fig. 1; Table 4).

**Table 4:** Number of the differentially expressed urinary lipids between the groups

	Kruskal–Wallis	Steel–Dwass^‡^		
FDR^†^	**HIC-BPS-control**	**HIC-BPS**	**HIC-control**	**BPS-control**
< 0.05	102	66	100	3
< 0.01	76	38	74	0
< 0.001	54	24	54	0
< 0.0001	43	13	42	0

^†^ FDR was set by the Benjamini–Hochberg correction

^‡^ Statistical significance of differences in urinary lipid levels was set at a *p*-value < 0.05 in the Steel–Dwass post hoc test and *log*_*2*_*[Ratio]* ≥ 1 in pairwise comparisons between groups

BPS: bladder pain syndrome; FDR: false discovery rate; HIC: Hunner-type interstitial cystitis

### Urinary lipid levels and disease classification test

Table 5 shows the top five urinary lipid species that distinguished patients with HIC from the other (BPS + control) groups, and the BPS from the control group, in the single lipid analysis.

**Table 5:** Urinary lipid species unique to patients with HIC or BPS

	Urinary lipid species	AUC	95% CI	Cut-off value (nM/g Cre)	Accuracy	Sensitivity	Specificity
HIC vs. others^†^	C24 Ceramide	0.792	0.731–0.858	44.233	72.2	64.7	81.7
	PC (34:1)	0.768	0.702–0.830	34.069	70.8	60.3	83.9
	PC (36:2)	0.765	0.699–0.829	11.118	72.7	72.4	73.1
	PC (36:3)	0.763	0.697–0.830	8.639	70.8	62.9	80.6
	PC (38:5)	0.761	0.697–0.827	3.482	72.7	69.8	76.3
BPS vs. control	LPC (14:0)	0.656	0.515–0.776	0.018	65.6	72.7	63.4
	PC (36:5)	0.610	0.501–0.712	0.643	47.3	86.3	35.2
	LPC (18:3)	0.588	0.480–0.698	0.030	48.3	81.8	38.0
	LPS (16:1)	0.571	0.470–0.662	0.551	39.7	90.9	23.9
	LPS (22:6)	0.563	0.413–0.716	0.721	68.8	50.0	74.6

^†^BPS and control

AUC: area under the curve; BPS: bladder pain syndrome; CI: confidence interval; HIC: Hunner-type interstitial cystitis; LPC: lysophosphatidylcholine; LPS: lysophosphatidylserine; PC: phosphatidylcholine

**Fig. 2:**
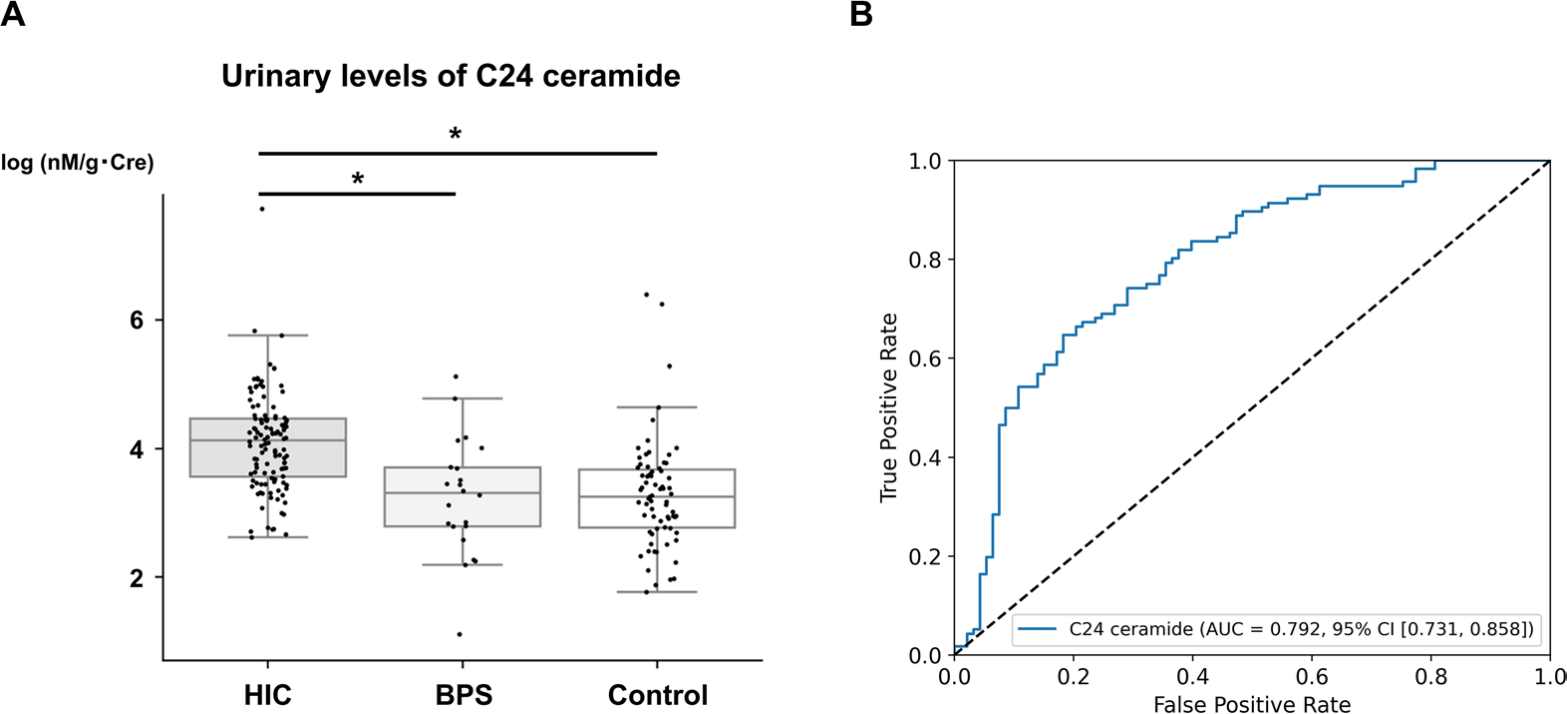
**A**: Box plots showing the log_2_-normalized urinary levels of C24 ceramide in patients with IC/BPS and in controls. Asterisks indicate statistically significant differences (*p* < 0.05). **B**: ROC curve analysis of the ability of urinary levels of C24 ceramide to discriminate HIC from others (BPS + controls). AUC: area under the curve, BPS: blader pain syndrome, CI: confidence interval, HIC: Hunner-type interstitial cystitis

Urinary levels of C24 ceramide were significantly higher in the HIC group than in the BPS and control group (Fig. 2A), with the highest AUC for diagnosing HIC being 0.792 (95% CI: 0.731–0.858), with an accuracy of 72.2%, a sensitivity of 64.7%, and a specificity of 81.7% at a cut-off value of 44.233 nM/g Cre (Fig. 2B). Meanwhile, LPC (14:0) showed the highest AUC (0.656 (95% CI: 0.515–0.776)) for discriminating those with BPS from the control, with an accuracy of 65.6%, a sensitivity of 72.7%, and a specificity of 63.4% at a cut-off value of 0. 018 nM/g Cre.

### Arithmetic operations on the paired lipids, and the disease classification test

Table 6 shows the five lipid pairs that, after performing the arithmetic operations, significantly discriminated HIC from other groups (BPS + control), and BPS from controls. The results indicated that the summed urinary levels of C24 ceramide and LPI (18:3) discriminated HIC from the other groups, with the highest AUC being 0.805 (95% CI: 0.745–0.867), and an accuracy of 74.2%, a sensitivity of 69.8%, and a specificity of 79.6% at a cut-off value of 43.115 nM/g Cre. Meanwhile, subtracting urinary levels of PG (36:5) from those of PC (38:2) had a highest AUC of 0.752 (95% CI: 0.639–0.848) for discriminating BPS from controls, with an accuracy of 73.1%, a sensitivity of 81.8%, and a specificity of 70.4% at a cut-off value of 1.778 nM/g Cre.

**Table 6:** Urinary lipid pairs for which arithmetic operations discriminated HIC from BPS

	Lipid pair	AUC	95% CI	Cut-off value (nM/g Cre)	Accuracy	Sensitivity	Specificity
HIC vs. Others^†^	C24 Ceramide + LPI (18:3)	0.805	0.745–0.867	43.115	74.2	69.8	79.6
	C24 Ceramide– PC (36:0)	0.804	0.746–0.865	41.544	72.7	62.9	84.9
	C24 Ceramide– PE (38:0)	0.798	0.739–0.863	31.422	74.6	80.2	67.7
	C24 Ceramide × PC (34:1)	0.798	0.738–0.858	1369.511	74.2	68.1	81.7
	C24 Ceramide + PC (34:1)	0.798	0.737–0.859	76.954	74.6	69.0	81.7
BPS vs. control	PC (38:2)– PC (36:5)	0.752	0.639–0.848	1.778	73.1	81.8	70.4
	Sph × PC (38:2)	0.743	0.631–0.837	161.947	72.0	77.3	70.4
	PC (38:2) × SM (38:3)	0.734	0.615–0.841	76.594	76.3	63.6	80.3
	PC (34:4) × PC (38:2)	0.733	0.616–0.833	145.593	70.4	72.7	70.4
	PC (38:2) × SM (38:4)	0.732	0.616–0.8335	805.673	74.1	72.7	74.6

^†^BPS and control

AUC: area under the curve; BPS: bladder pain syndrome; CI: confidence interval, HIC: Hunner-type interstitial cystitis; LPI: lysophosphatidylinositol, PC: phosphatidylcholine; PE: phosphatidylethanolamine, Sph: sphingosine; SM: sphingomyelin

### Machine learning models and the disease classification test

**Fig. 3:**
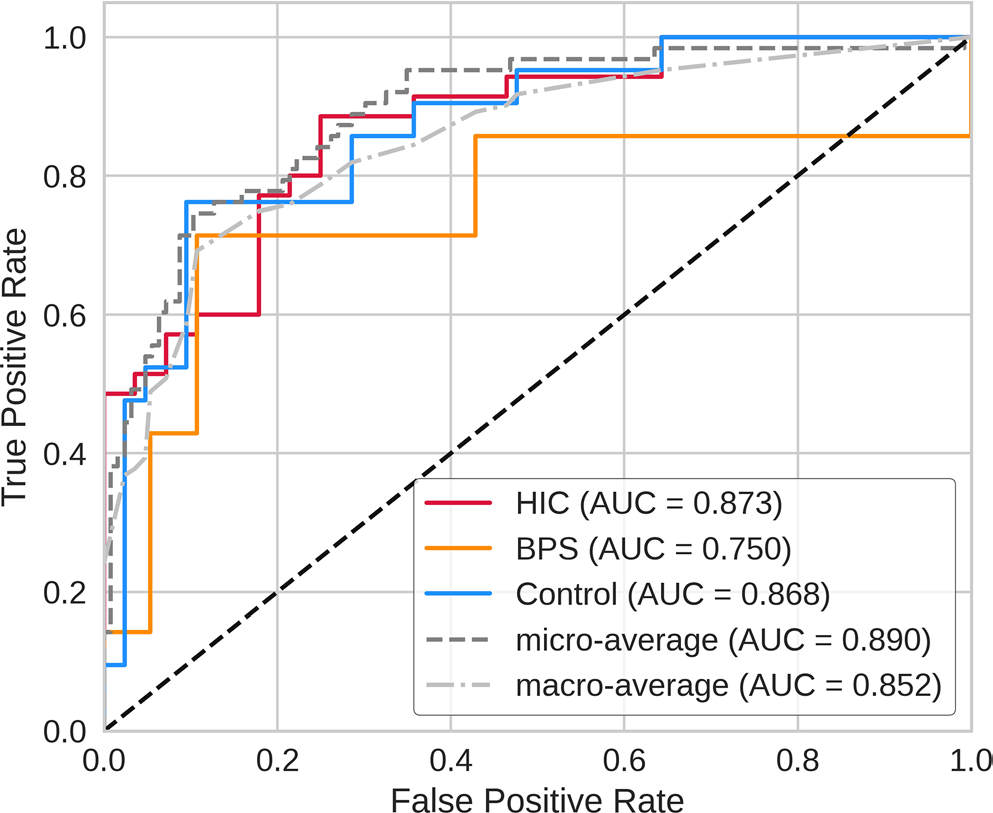
ROC curves for the constructed machine learning model for classification of IC/BPS and controls. The model was constructed by inputting urinary levels of whole lipid species along with patients’ age and sex. Red, orange, and blue represent the ROC curves for HIC, BPS, and controls, respectively. The black and gray dotted lines are the ROC curves for the micro and macro average AUC. AUC: area under the curve, BPS: blader pain syndrome, CI: confidence interval, HIC: Hunner-type interstitial cystitis

Urinary lipid data from all of the study subjects (*n* = 209) were assigned randomly to a training dataset (*n* = 146, including 81 HIC samples, 15 BPS samples, and 50 control samples) for machine learning, and a test dataset (*n* = 63, including 35 HIC samples, seven BPS samples, and 21 control samples) for external validation. We constructed eight machine learning models to account for variations in the input datasets, which comprised whole urinary lipids or selected DELs based on the FDR from the Kruskal–Wallis test (Table 4): Models 1 and 2, 218 lipid species with (Model 2) and without (Model 1) demographic data (age and sex); Models 3 and 4, 102 lipid species (FDR < 0.05 in the Kruskal–Wallis test) with (Model 4) and without (Model 3) demographic data; Models 5 and 6, 76 lipid species (FDR < 0.01) with (Model 6) and without (Model 5) demographic data; and Models 7 and 8, 54 lipid species (FDR < 0.001) with (Model 8) and without (Model 7) demographic data. Table 7 shows the diagnostic performance of the eight constructed models for the test dataset. The model constructed using all 218 lipid species plus patient age and sex showed the highest diagnostic accuracy for IC/BPS, with an AUC of 0.873 for HIC and an AUC of 0.750 for BPS (Fig. 3).

**Table 7:** Diagnostic performance of the machine learning model based on urinary lipidomic data and patient demographics

Input data					AUC		
Lipid	Sex, age	Accuracy	HIC vs. others^†^	BPS vs. others	Control vs. others	Micro-average	Macro-average
218	**-**	65.1	0.819	0.584	0.768	0.824	0.734
	**+**	74.6	0.873	0.750	0.868	0.890	0.852
102	**-**	63.5	0.831	0.617	0.774	0.832	0.753
	**+**	76.2	0.864	0.704	0.840	0.869	0.812
76	**-**	69.8	0.846	0.653	0.789	0.841	0.774
	**+**	71.4	0.818	0.571	0.822	0.838	0.753
54	**-**	66.7	0.821	0.589	0.761	0.820	0.737
	**+**	73.0	0.861	0.469	0.840	0.852	0.736

^†^BPS and control

AUC: area under the curve; BPS: bladder pain syndrome; HIC: Hunner-type interstitial cystitis

## Discussion

In the present study, we performed machine learning coupled lipidomic analysis of urine samples from patients with IC/BPS and from control patients, and identified unique urinary lipid signatures in patients with IC/BPS. We observed that (1) urinary levels of C24 ceramide distinguished HIC from BPS and controls (single urinary lipid analysis); (2) summed urinary levels of C24 ceramide and LPI (18:3) distinguished HIC from BPS and controls more accurately than C24 ceramide alone; and subtraction of urinary PC (36:5) levels from those of PC (38:2) distinguished BPS from controls (paired urinary lipid analysis with arithmetic operations); and (3) a machine learning model constructed using all detected urinary lipids and subject demographics could distinguished IC/BPS more accurately than either single or paired lipid analyses, with an AUC of 0.873 and 0.750 for HIC and BPS, respectively.

Ceramide, a sphingolipid composed of a sphingosine amide linked to a fatty acid, plays an important bioactive role in cellular signaling and protein translocation through the cell membrane, especially during induction of apoptosis [21]. Studies report that ceramide is involved in various pathological conditions, including inflammation [11, 22]. Formation of C24 ceramide is enhanced in response to stress stimuli, and induces a multitude of changes with respect to organization and fluidity of cell membranes, leading to activation of a series of cellular biological processes such as inflammatory reactions [23]. Considering that urinary levels of C24 ceramide are also elevated in cases of acute bacterial cystitis [14], it may be a common lipid mediator of inflammation and not specific to the HIC inflammation. Further investigations into the biological role of C24 ceramide and related lipid molecules in the pathophysiology of HIC, in conjunction with comparing the urinary levels of these lipid molecules between HIC and other chronic inflammatory conditions of the bladder, are needed.

IC/BPS collectively impairs QOL because of persistent, intractable bladder pain and lower urinary tract symptoms. Specifically, HIC deforms the bladder structure and gradually reduces bladder capacity as the disease progresses, whereas BPS rarely affects bladder tissue and capacity [24]. Therefore, it is crucial to accurately distinguish HIC from other forms of IC/BPS at an early stage and manage it with optimal anti-inflammatory treatments to preserve bladder capacity as much as possible during long-term clinical management [25]. At present, however, there are no reliable, minimally invasive diagnostic markers for HIC. Results of the present study suggests that urinary levels of C24 ceramide alone, or arithmetically calculated C24 ceramide and LPI (18:3) levels, could discern HIC from the IC/BPS umbrella. Future studies to test the validity and reproducibility of these candidate lipids as reliable indicators for HIC in the real-world setting are warranted.

With respect to BPS, the highest diagnostic accuracy from the single lipid and paired lipid analyses reached an AUC of only 0.656 (based on LPC (14:0)) and 0.752 (based on PC (38:2) minus PC (36:5)), respectively. These results may reflect the phenotypic features of IC/BPS: HIC is a distinct entity, whereas BPS is a more complex syndrome that encompasses miscellaneous conditions. Thus, urinary lipid signatures in patients with HIC may be distinct from those in patients with other IC/BPS phenotypes.

Our study also suggests the future possibility that AI-driven models can form the basis of flagship diagnostic systems for IC/BPS, as for other diseases (e.g., endoscopic diagnosis of gastrointestinal diseases). Recently, we developed deep learning models that recognize cystoscopic Hunner lesions much more accurately than expert human IC/BPS urologists, with a mean AUC of 0.919 [26]. Here, the machine learning-coupled urinary lipidomics model showed the best diagnostic performance for HIC (AUC = 0.873), exceeding that of the single and paired urinary lipid analyses. These results suggest that machine learning-coupled urinary lipidomics analysis, along with the deep learning models of cystoscopic recognition of Hunner lesions, could be the next-generation of AI-driven models for diagnosis of HIC. Future studies using other independent cohorts for external validation of our machine learning-coupled urinary lipidomics model for IC/BPS are required.

This study has several limitations. First, the timing of urine sample collection was not standardized. This means that levels of urinary lipid species might have been analyzed in some symptomatic patients, while others were asymptomatic at the time, especially in patients with IC/BPS, which might affect the levels of responsible urinary lipid molecules in patients with IC/BPS and thus biased the results. However, we compensated for this by collecting as many samples as possible, resulting in enrollment of 200 cases. Second, the number of BPS patients was relatively small when compared with the numbers of HIC and control patients, which might affect the classification performance of the machine learning model. Third, there were differences in the age and sex ratios between the three groups. Although our previous study reported no significant differences in urinary lipid signatures according to sex [14], this difference may have affected the results of the present study. Last, the opaque black box nature of the machine learning process and diagnostic nature of a Hunner lesion as warranted assertibility could be other limitations to the methodology.

## Conclusions

Urinary levels of C24 ceramide alone, or arithmetically calculated C24 ceramide and LPI (18:3) levels, may be potential biomarkers for distinguishing patients with Hunner lesions from those without. Machine learning-coupled urinary lipidomics may contribute to constituting the next-generation diagnostic systems for interstitial cystitis.

## Electronic supplementary material

Below is the link to the electronic supplementary material.

Supplementary Material 1

## Data Availability

No datasets were generated or analysed during the current study.
