# Supplementary material for: Lipidomic analysis coupled with machine learning identifies unique urinary lipid signatures in patients with interstitial cystitis/bladder pain syndrome
Source: World J Urol. 2025 Apr 18;43(1):233. doi: 10.1007/s00345-025-05628-y (PMC12008056; doi:10.1007/s00345-025-05628-y)
Supplement: Supplementary file 1 — Supplementary Material 1 [file 345_2025_5628_MOESM1_ESM.docx]

**Lipidomic analysis coupled with machine learning identifies unique urinary lipid signatures in patients with interstitial cystitis/bladder pain syndrome**

Takuya Iwaki^1,2^, Makoto Kurano^3*^, Masahiko Sumitani^4^, Aya Niimi^1^, Akira Nomiya^5^, Jun Kamei^1^, Satoru Taguchi^1^, Yuta Yamada^1^, Yusuke Sato^1,6^, Masaki Nakamura^7^, Daisuke Yamada^1^, Tomonori Minagawa^8^, Hiroshi Fukuhara^9^, Haruki Kume^1^, Yukio Homma^10^, and Yoshiyuki Akiyama^1,8*^

^1^Department of Urology, Graduate School of Medicine, The University of Tokyo, Tokyo, Japan

^2^Department of Urology, Chiba Tokushukai Hospital, Chiba, Japan

^3^Department of Clinical Laboratory Medicine, The University of Tokyo, Tokyo, Japan

^4^Department of Pain and Palliative Medicine, The University of Tokyo Hospital, Tokyo, Japan

^5^Department of Urology, Japan Organization of Occupational Health and Safety, Kanto Rosai Hospital, Kanagawa, Japan

^6^Department of Urology, Tokyo Metropolitan Tama Medical Center, Tokyo, Japan

^7^Department of Urology, NTT Medical Center Tokyo, Tokyo, Japan

^8^Department of Urology, Shinshu University School of Medicine, Nagano, Japan

^9^Department of Urology, Kyorin University School of Medicine, Tokyo, Japan

^10^Department of Interstitial Cystitis Medicine, Kyorin University School of Medicine, Tokyo, Japan

*Address reprint requests and correspondence to:

Yoshiyuki Akiyama, M.D., Ph.D. (lead contact)

Department of Urology, Shinshu University School of Medicine, Nagano, Japan

E-mail: yoshiyuki-akiyama@shinshu-u.ac.jp

Telephone: +81-26-337-2661 (0263-37-2661); FAX: +81-26-337-3082 (0263-37-3082)

ORCID: 0000-0003-1439-5640

**Supplement**

***Sample collection and patient demographics***

Spot midstream urine samples were collected from 116 patients with HIC, 22 patients with BPS, and 71 control patients; samples were obtained during outpatient visits to the urology clinic at the University of Tokyo Hospital from September 2020 to March 2021. Patient symptoms (at the time that the samples were collected) were assessed using the IC/BPS symptom scores measured by the O’Leary and Sant symptom index (OSSI) and problem index (OSPI) [16]; an 11-point numerical rating scale of pain intensity, with 0 indicating no pain and 10 indicating the worst pain ever; a frequency volume chart including the daytime and nighttime urinary frequency; and the average and maximum voided volume. All patients with HIC had been treated with transurethral resection of Hunner lesions with concomitant bladder hydrodistension, and were regularly followed up during outpatient visits. These patients managed symptom flairs using only analgesia and antibiotics. Patients with BPS were treated with oral medications on an outpatient basis. None of the patients with IC/BPS had received intravesical therapies before enrollment. Control urine samples were obtained from 71 patients with benign urological diseases (n=28) or a previous history of urological malignancy (n=43); samples were obtained at outpatient visits (as for patients with IC/BPS). All control patients were free from bladder pain and discomfort. Of the 28 patients with benign urological diseases, 13 had an overactive bladder, nine had benign prostatic hyperplasia, two had upper tract urolithiasis, one had radiation-induced cystitis, one had allergic cystitis, one had bladder amyloidosis, and one had asymptomatic lupus cystitis; regardless of medication status, all were followed up regularly. Of the patients with previous urological malignancy, 21 had a previous history of prostate cancer (15 had undergone radical prostatectomy and six had undergone radiation therapy); 15 patients had non-muscle invasive bladder cancer (nine had undergone transurethral resection of the tumor alone, and six had received adjuvant intravesical treatment with mycobacteria bacillus Calmette-Calmette-Guérin after transurethral resection of the tumor); three patients had renal cell carcinoma (treated with radical nephrectomy); three had pheochromocytoma (treated with adrenalectomy); and one had testicular cancer (treated with high orchiectomy without adjuvant chemotherapy). All were followed up regularly. Collected urine samples were stored at -80°C until measurement were conducted, and all samples were handled on ice during the procedures (except during necessary incubation processes).

***Statistical Analysis***

Statistical analyses of urinary lipid data processing and machine learning analyses were conducted using the Scikit-learn library in Python [20]. Other statistical analyses were carried out using JMP® Pro software, ver. 11 (SAS Institute, Cary, NC). Fisher’s exact test was used in both two-group and three-group comparisons for categorical variables. Wilcoxon rank sum test in the two-group comparisons and the Kruskal–Wallis test followed by the Steel–Dwass post hoc test in the three-group comparisons were used for continuous variables, respectively. The significance level was set at *p* <0.05.
